# Spatial working memory is enhanced in children by differential outcomes

**DOI:** 10.1038/srep17112

**Published:** 2015-11-24

**Authors:** Laura Esteban, Ana B. Vivas, Luis J. Fuentes, Angeles F. Estévez

**Affiliations:** 1 Departamento de Psicología, Universidad de Almería, Almería, Spain; 2 Psychology Department, The University of Sheffield International Faculty, City College, Thessaloniki, Greece; 3 Departamento de Psicología Básica y Metodología and Regional Campus of Excellence “Mare Nostrum”, Universidad de Murcia, Murcia, Spain

## Abstract

Working memory (WM) is essential to academic achievement. Any enhancement of WM abilities may improve children’s school performance. We tested the usefulness of the differential outcomes procedure (DOP) to enhance typically developing children’s performance on a spatial WM task. The DOP involves a conditional discriminative learning task in which a correct choice response to a specific stimulus-stimulus association is reinforced with a particular reinforcer (outcome). We adapted a spatial memory task to be used with the DOP. Participants had to learn and retain in their WM four target locations of eight possible locations where a shape could be presented. Two groups of 5- and 7-year-old children performed the low-attentional version of the spatial task, and an additional group of 7-year-old children performed the high-attentional version. The results showed that compared with the standard non-differential outcomes procedure (NOP), the DOP produced better memory-based performance in 5-year-old children with the low-attentional task and in 7-year-old children with the high-attentional task. Additionally, delay intervals impaired performance in the NOP but not in the DOP. These findings suggest that the DOP may be a useful complement to other WM intervention programs targeted to improve children´s academic performance at school.

Working memory (WM) has been conceptualized as a specialized multi-component system that is associated with different aspects of cognitive activity^1^,^2^,^3^. It is crucial to a variety of higher cognitive functions, such as reading, language comprehension, mathematical abilities and reasoning^4^,^5^,^6^, and to academic attainment^7^. In Baddeley’s influential WM model, the central executive has the critical role of controlling how information from different stores is both manipulated and integrated according to task demands. The central executive system is complemented by the existence of some memory stores that hold information either in a phonological code (the phonological loop) or in a visuo-spatial code (the visual-spatial sketchpad). The latter is involved in both the holding and manipulating of visual and spatial information under the control of the central executive.

In the present study, we focused on the spatial component of WM. Spatial memory is vital for survival. It allows animals to find food and enables humans to navigate around the city or simply find their possessions. It also permitted our ancestors to survive in hostile environments by facilitating the finding of food, water and shelter. Individuals must not only remember where objects are located but must frequently do so while performing other demanding activities. In those cases, not only is the retention of locations necessary, but holding and manipulating information in one’s mind for short periods of time while concurrently inhibiting distracting information are also required.

Within the context of school, the spatial component of WM, rather than the visual, seems to be the determinant factor in achieving better academic performance^8^. Thus, children exhibiting poor performance on spatial WM tasks but not visual ones also tend to receive low scores on problem-solving tasks^9^. Moreover, this relation is more apparent in spatial tasks with high attentional demands, that is, tasks that tax the executive control system more than those that only require passive recall of information^10^.

Recently, WM training has been shown to enhance children’s learning abilities at school^11^; hence, any procedure that facilitates those abilities has the potential to improve academic performance. Our previous research has shown that memory for abstract discriminative learning produces long-term persistence and resistance to forgetting when a differential outcomes procedure (hereafter, DOP) is implemented^12^,^13^. The DOP requires the structure of a conditional discriminative learning task in which a correct choice response to a specific stimulus-stimulus association is reinforced with a particular reinforcer or outcome^14^,^15^. Conditional discrimination tasks consist of the presentation of a sample stimulus that is associated with one of several comparison stimuli. Each particular sample-comparison stimulus association requires a specific response that must first be learned and then reinforced with a unique outcome (see Trapold, 1970 for the first demonstration of the DOP in rats). When the reinforcement of correct responses is arranged according to the DOP rather than the more standard non-differential outcomes procedure in which the reinforcers are randomly administered, the rate of acquiring the association accelerates, and the final accuracy level is higher.

The DOP has been widely proven to enhance not only conditional discriminative learning but also memory in both animals and humans (for reviews^16^,^17^,^18^). There is a bulk of evidence demonstrating improvement of memory-based performance in healthy people (children^19^,^12^,^13^; younger adults^20^,^21^; and older adults^22^) and in people with various pathologies (e.g., Down syndrome^23^; dementia^24^; Korsakoff’s syndrome^25^; Prader-Willi syndrome^26^). The DOP effect has been accounted for in terms of expectancies functioning as prospective memory representations elicited by the to-be-memorized stimuli for which the outcomes will be forthcoming^18^. Expectancies about the forthcoming outcome provide an additional source of information so that performance is less affected by delays or working memory demands. Moreover, a recent study demonstrated that in some situations, expectancies as cues for guiding choice behavior in symbolic conditional discrimination tasks may be even more powerful than the discriminative stimuli themselves^27^. At the neural level, Ramirez and Savage^28^ showed that the basolateral amygdala and the orbitofrontal cortex are critical for the development and maintenance, respectively, of outcome expectancies when hedonic reinforcers are used in animals. In human research, Mok, Thomas, Lungu, and Overmier^29^ observed increased delay-period activity in sensory-specific areas that depend on the outcome modality (visual or auditory). These sensory-specific activations were complemented with non-modality-specific activation in the posterior parietal cortex mediating delay-period expectations. Thus, the results from both animal and human studies suggest that the DOP recruits brain areas involved in prospective memory irrespective of the modality used for reinforcement. In contrast, under more standard non-differential outcome conditions, there is only one source of information that can guide correct responding. Participants must then rely on their memory of the previously presented sample stimulus to solve the task successfully^29^,^18^. The hippocampus has been mainly involved in that process in both animals^30^ and humans^29^. Mok *et al.*^29^ also observed that the DOP benefit derived from an early transition from retrospection to prospection that activated a more enduring long-term associative memory.

In agreement with the results obtained by Mok *et al.*^29^ we showed that typically developing children exhibited long-lasting effects of conditional discriminative learning that remained after subsequent follow-up over a month later^12^,^13^. Importantly, this long-lasting effect was observed in children aged 5^12^ and 7 years^13^, with both primary and secondary reinforcers and different types of reinforcement (children received a reinforcer following a correct choice; a reinforcer was removed after an incorrect choice; or the combination of both). These findings suggest that the DOP is a robust procedure that can be easily implemented in diverse learning contexts. However, although a long-lasting memory of previous learning is essential for optimal academic achievement, WM functioning more accurately predicts children’s performance at school, particularly in mathematics and reading, independent of children’s general intellectual abilities^6^,^7^,^31^,^32^.

Despite the relevance of WM in children’s learning process, the DOP has not yet been used in the context of spatial WM. In the present study, we investigated whether the DOP is also useful to enhance spatial WM performance in typically developing children. If that were the case, the DOP might be an excellent complement to other training interventions because it consists simply of arranging outcomes (feedback) in such a way that they are unique to each correct stimulus-choice response. As previously mentioned, DOP has been shown to both accelerate learning and increase final accuracy.

We designed two spatial memory tasks with two levels of attentional demands. One task simply required keeping track of the location of sequentially presented stimuli, with minimal demands of the central executive (the low-attentional task). The other task was similar to the previous one but included a secondary task that required a prompt response and thus taxed the central executive (the high-attentional task). We ran the low-attentional spatial WM task with both 5- and 7-year-old children and the high-attentional task with an additional group of 7-year-old children. The younger children only ran the low-attentional task because previous research has determined the age of 6 years to be the development point at which the full structure of working memory is established^33^. That is, WM structures do not seem to be fully developed until 6 years of age, which is consistent with the incomplete maturation of the prefrontal and parietal brain regions on which WM functions are highly dependent^34^,^35^. Consequently, even the low-attentional version of the spatial WM task may be highly demanding for that age. For older children, the WM structures should be fully developed; therefore, only the high-attentional task is expected to cause detrimental performance compared with the low-attentional task. Because the DOP is particularly effective when learning is more taxing and attentionally demanding^21^,^36^,^37^,^38^, we expected that it would enhance the spatial WM in the younger children when they performed the low-attentional task and in the older children when they performed the high-attentional task. For the older children, performance should deteriorate with the high-attentional task compared with the low-attentional task, but to a lesser extent with the DOP than with the standard non-differential outcomes procedure (hereafter, the NOP).

## Results

A first analysis was conducted on the low-level version of the task. Accuracy data were submitted to a 4 × 2 × 2 mixed ANOVA, with Delay (1, 5, 10 and 15 seconds) and Outcomes (DOP and NOP) as the within-participant factors and Age (younger and older children) as the between-participant factor. A second analysis was conducted with the other group of 7-year-old children, who performed the high-attentional task only. Accuracy data were submitted to a 4 × 2 repeated measures ANOVA, with Delay (1, 5, 10 and 15 seconds) and Outcomes (DOP and NOP) as within-participant factors. We also assessed the differences between the low- and high-attentional tasks with the data provided by the two groups of 7-year-old children. Because the pattern of results was similar for boys and girls, data were collapsed across sex. Statistical analyses were performed using SPSS v22.0.

### Effects of age and outcomes procedure in the low-demand task

**Figure 1:**
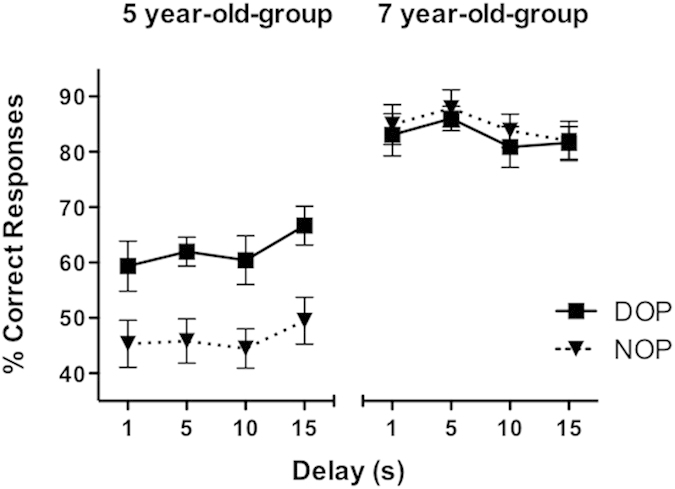
Mean percentage of correct responses as a function of Age (younger and older children), Delay (1, 5, 10 and 15 seconds), and Outcomes (differential and non-differential) on the low-attentional task. Error bars represent the mean standard errors.

The results are illustrated in Fig. 1. The analysis conducted on the percentages of correct responses showed significant main effects of Outcomes [*F*(1, 27) = 8.69, *p* = 0.007, η_p_^2^ =0. 244] and Age [*F*(1, 27) = 108.7, *p* < 0.001, η_p_^2^ = 0.801]. That is, participants showed better performance when differential outcomes were arranged (72% vs. 65% for the DOP and NOP, respectively), and older children were more accurate than younger children were (84% vs. 54%, respectively). The Outcomes x Age interaction was also significant [*F*(1, 27) = 13.54, *p* = 0.001, η_p_^2^ = 0.334]. The analysis of the interaction revealed a significant simple main effect of Outcomes for the younger group (62% vs. 46% for the DOP and NOP, respectively) [*F*(1, 11) = 17.84, *p* = 0.001, η_p_^2^ = 0.619], but not for the older group (83% vs. 85% for the DOP and NOP, respectively) [*F* < 1]. The performance of 5-year-old children was at chance with the NOP (Chi-square = .25, df = 1; *p* > 0.05), but it was above chance with the DOP (Chi-square = 4.0, df = 1; *p* < 0.05; given that in the low-demand task the probe display contained only two choice stimuli, the chance level was at 50% accuracy). Neither the main effect of Delay nor its interactions with the other two factors was statistically significant (all *p*s > 0.05).

### Effect of outcomes as a function of task demands

**Figure 2:**
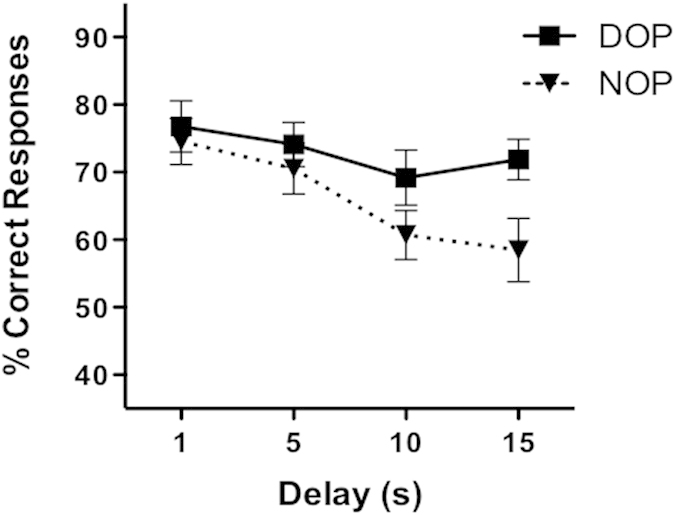
Mean percentage of correct responses as a function of Delay (1, 5, 10 and 15 seconds) and Outcomes (differential and non-differential) on the high-attentional task. Error bars represent the mean standard errors.

In contrast to the results with the low-attentional task, 7-year-old children showed a main effect of Outcomes with the high-attentional task [*F*(1, 13) = 7.06, *p* = 0.020, η_p_^2^ = 0.352]. The performance was higher with the DOP (73%) than with the NOP (66%). We also observed a main effect of Delay (76%, 72%, 65%, and 65% for delays 1, 5, 10, and 15 seconds, respectively) [*F*(3, 39) = 6.0, *p* = 0.001, η_p_^2^ = 0.316], although the effect was due to higher performance with the two short delays combined (Mean = 74%) compared with the two long delays combined (Mean = 65%) [*F*(1,13) = 12.08, *p* = 0.004, η_p_^2^ = 0.482]. However, the difference between the short and long delays was observed for the NOP (73% vs. 60%) [*t*(13) = 3.43; *p* < 0.01] but not the DOP (75% vs. 71%) [*t*(13) = 1.87; *p* > 0.05]. The differences between delays as a function of the outcomes procedure were further qualified by the emerging marginally significant interaction between the factors [*F*(1, 13) = 4.09, *p* = 0.064, η_p_^2^ = 0.239]. The interaction was due to a significant effect of Outcomes just for the long delays [*F*(1, 13) = 12.42, *p* = 0.004, η_p_^2^ = 0.489]. The same pattern of results was found when the four delays were incorporated into the analysis. The linear trend was significant for the NOP [*F*(1, 13) = 12.92, *p* < 0.003, η_p_^2^ = 0.498] but not the DOP (*p* > 0.05) (Fig. 2). Participants showed significantly better performance in the DOP condition only when the two longer delays were analyzed [*F*(1, 13) = 4.84, *p* = 0.046, η_p_^2^ = 0.271 and *F*(1, 13) = 5.10, *p* = 0.042, η_p_^2^ = 0.282 for delays 10 and 15 sec, respectively].

In the final analysis, we compared the performances of the two 7-year-old groups to assess the detrimental effects of the high-attentional task relative to the low-attentional task. The main effect of Task was significant [*F*(1, 29) = 18.92, *p* < 0.001 η_p_^2^ = 0.395], which indicated that the high-attentional task impaired children’s performance compared with the low-attentional task. Importantly, the Outcome (DOP, NOP) × Task type (low-, high-attentional) interaction was significant [*F*(1, 29) = 4.5, *p* = 0.043, η_p_^2^ = 0.134]. To further analyze this interaction, we first computed a low-attentional performance score by combining both the two outcomes procedures and the four delays because these two factors did not produce any difference with such version of the task. The average score on the low-attentional task was 84% correct. When compared with such a low-attentional task score, the high-attentional task showed a decrement in performance with both the DOP (84% vs. 73%) [*F*(1, 29) = 11.54, *p* = 0.002, η_p_^2^ = 0.285] and the NOP (84% vs. 66%) [*F*(1, 29) = 22.17, *p* < 0.001, η_p_^2^ = 0.433], but the detriment was smaller with the DOP than with the NOP (11% vs. 18%) [*F*(1, 13) = 7.06, *p* = 0.020, η_p_^2^ = 0.352].

## Discussion

In recent years, there has been a spectacular increase in the use of training programs that attempt to enhance children’s school performance. Some programs have focused on training those abilities with which children show special difficulties. For instance, Dowker and her colleagues have developed several training programs that use exercises similar to those that form part of specific learning and are thus based on repetition and practice. Thus, to improve mathematical abilities, they developed the Numeracy Recovery and the Catch-Up Numeracy programs^39^,^40^ and implemented the Catch-Up Literacy program to improve reading abilities^41^. Other intervention programs focus on the cognitive processes that are thought to be crucial for learning, such as executive functions (see Diamond & Lee, 2011^42^ for review), the executive attention network^43^, or more specifically, WM^11^.

Although many intervention programs have probed their efficiency when training those processes, they usually require a series of computerized exercises that children perform in a variable number of sessions lasting several weeks or months^11^. In the present study, we approached the enhancement of WM from a different perspective that might complement the aforementioned ones. Specifically, we probed the benefit for children’s spatial WM memory of a procedure (the DOP) that was imported from animal studies and has been widely used in discrimination learning and memory-based tasks with both humans and animals (see López-Crespo & Estévez, 2013^17^ for a review; see Williams *et al.* 1990^44^ for an example of the DOP being used to enhance spatial working memory in animals).

The main findings of the present study can be summarized as follows. On the low-attentional version of the spatial WM task, only the younger children benefited from the DOP arrangement. In fact, younger children performed at chance with the standard non-differential outcomes condition; that is, they were unable to keep in memory the four potential target locations in an eight-location spatial array. However, when each target location, marked by the relevant shape, was associated with a unique outcome (the DOP condition), their performance was above chance, which indicated that they could retain in memory where the target locations were positioned with certain accuracy. For the older children, the low-attentional task was very easy, and they could retain the four target locations in memory with high accuracy, regardless of the outcome manipulation. When we modified the spatial WM task to include both a secondary task and an additional irrelevant shape, the attentional demands increased considerably, which taxed the central executive more than the low-attentional task. The children exhibited a clear detriment in performance, although it was more apparent with the NOP than with the DOP. Again, the DOP arrangement improved performance compared with the NOP, particularly with longer delays.

The expectancy theory outlined in the introduction can explain why the DOP arrangement was beneficial to the children in the present study. At the initial stage of learning, the memory of whether the shape marked a target location is highly dependent upon the retrospective recall of the target locations, which is a process that seems to rely on the hippocampus^29^. As the learning of target locations progressed in a trial-and-error fashion, children were able to activate a prospective representation (expectative) of the forthcoming outcome associated with each target location. The presence of the shape at the target location at the time of the memory test could have functioned as a cue that activated the unique outcome representation (expectancy), thereby facilitating memory performance. Participants’ responses in the DOP condition may have then moved from being based only on retrospection to being based on prospection as well. As previously mentioned, prospective memory is based on different neural mechanisms from those involved in retrospective memory and is less affected by working memory demands and delays. In contrast, under the NOP condition, the retrospective recall of the four target locations was the only available source of information to guide children’s behavior throughout the experiment.

It is worth noting that when the memory task involves more than the passive recovery of information, the executive frontal system is also involved, although tasks can vary in how much the central executive is taxed^45^. The two-year age difference between our participant groups had enormous consequences for memory performance. In the NOP condition, 5-year-old children were unable to retain information and scored no better than chance. However, performance was much better (above 80% accuracy) in 7-year-old children. These results are consistent with the notion that age 6 represents an inflexion point in development where the WM structures linked to the frontal lobe become fully functional^33^. It may also explain why the benefit of the DOP with the younger children was rather modest and why a delay effect was not observed in that age. If the ability to retain in memory the expectations about the forthcoming outcome depends also on frontal brain regions, as previously observed in animals^28^, then those structures might still be immature at that age and might have impaired performance on the memory task, even when differential outcomes were arranged. However, it may also be argued that the training session was rather short; thus, more trials/sessions might have had a greater impact on performance (this is also valid for the high-attentional task). A task for future studies may be to test that possibility.

Finally, an additional benefit of the DOP was that the delay manipulation did not affect performance in either the younger or older children. These results further support our previous observations of children from the same ages when conditional discrimination learning was tested one day, one week or one month after training^12^,^13^. Performance was greatly affected, especially by delay, when learning took place with the NOP, but it was virtually unaffected when we used the DOP. This resistance to forgetting may have important implications for using the DOP in more applied contexts. The DOP is a simple procedure that can be easily set up to complement other training interventions. The present findings clearly show that the memory of contents acquired through the DOP lasts longer and is more resistant to forgetting.

## Methods

### Participants

Forty-three typically developing children (22 boys and 21 girls) participated in the experiment. Children ranged in age from 4 years and 2 months to 8 years and 10 months and were recruited from a public school (C.E.I.P. Lope de Vega) in Almería, Spain. Participants were assigned to two groups according to age. The younger (5 years) group (N = 12) completed only the low-attentional task. The older (7 years) group (N = 31) was split into two subgroups: 17 children completed the low-attentional task, and 14 children performed the high-attentional task. All children had normal or corrected-to-normal vision, and none had any evidence of learning difficulties. The experimental protocols were approved by the Ethics Committee of the University of Almeria, and the study was performed in accordance with the approved guidelines and the Declaration of Helsinki. Parental informed consent was obtained for each child who participated.

### Stimuli and materials

Because the outcome factor was manipulated within-subjects, we designed two versions (A and B) for both the low- and the high-attentional spatial WM tasks, one to be used with the DOP and the other with the NOP, counterbalanced across participants. The two versions of each task differed only in the geometric shape that marked the target and non-target locations. In version A, the shape was a 2.5 × 2.5 cm white square, whereas in version B, the shape was a 5 × 2.5 cm lime rectangle. The stimuli were presented on a black background on a touch screen (12.1” TFT LCD WXGA monitor) located on a child-size table. The E-prime program^46^ controlled the stimulus presentation as well as data collection. The shapes could appear in one of eight positions arranged in a 3 × 3 imaginary rectangle equidistant from the borders.

The primary reinforcers were candies, lollipops, stickers and pencils. Pictures of the primary reinforcers were used as immediate secondary reinforcers in the task (i.e., the outcomes). The pictures were presented individually at the center of the screen following a correct choice response. The reinforcers were selected because of their attraction to both younger and older children. At the end of the experiment, all children, regardless of their performance, received at least two hedonic outcomes (primary reinforcers) along with verbal appraisal.

Before the experimental sessions, the Peabody Picture Vocabulary Test-III (PPVT-III^47^) and the Automated Working Memory Assessment (AWMA^48^) were administered to each participant. The PPVT-III is a test of receptive vocabulary that provides a quick estimate of verbal ability and scholastic aptitude. The test lasted for approximately 15 minutes and consisted of a series of pictures (four numbered pictures to a page). Children were asked to point to one of the four pictures that corresponded to the word spoken by the examiner. The AWMA is a computer-based assessment of working memory skills. Verbal and visuo-spatial working memory are measured using tasks involving the simultaneous storage and processing of information, whereas tasks involving only storage of information are used to assess verbal and visuo-spatial short-term memory. We employed the short form that consisted of four tests: listening recall (verbal working memory), digit recall (verbal short-term memory), dot matrix (visuo-spatial short-term memory), and spatial recall (visuo-spatial working memory). The AWMA functions as a screening instrument for children with significant memory problems and yields a detailed profile and provides age-related cut-off scores that are indicative of typically low, average or high memory skills.

### Procedure

Each participant sat next to the experimenter in a quiet room at the school. The experiment consisted of two phases. In the first phase (the assessment phase), the experimenter assessed participants’ mental age and WM skills by administering the PPVT-III and the AWMA, respectively. All participants showed a mental age that was equal to or higher than their chronological age as well as AWMA standard scores within the average-to-high range (see Table 1), which indicated that the children in the sample had good working memory skills.

In the second phase (the experimental phase), two separate 30-minute experimental sessions were scheduled. In the first session, participants were randomly assigned to one of the two outcomes procedures (DOP or NOP). Each child then performed the task under the other outcomes procedure a week later. Thus, participants performed one of the two versions of the spatial memory task (e.g., version B) under one training condition (e.g., the NOP) with one set of four to-be-remembered target locations; one week later, they performed the other version of the task (e.g., version A) under the other training condition (e.g., the DOP) using the other set of four target locations. In each version of the task, four locations were never used as target locations (the non-target locations); therefore, any response to the shape located to one of those locations was never reinforced. In the DOP condition, each target location was always associated with a specific outcome (e.g., correct responses to the shape appearing on the right upper corner of the screen was always followed by the picture of lollipops). In the NOP condition, correct responses to shape locations were followed by a random presentation of a picture corresponding to one of the four secondary reinforcers. Thus, each target location in the NOP condition was equally often paired with each of the four pictures of the primary reinforcers.

**Figure 3:**
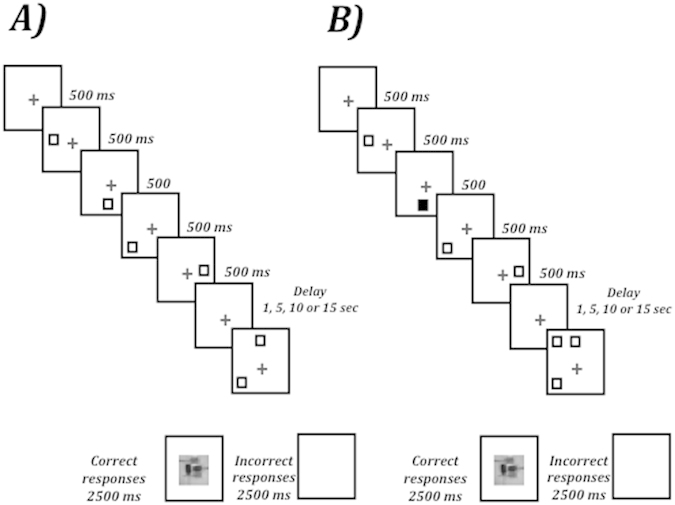
Sequence of stimuli and exposition durations used in the spatial memory task. (**A**) low-attentional task. (**B**) high-attentional task.

At the beginning of each experimental session, the experimenter verbally explained the task to each participant while a sample trial was shown on the screen. Then, participants were required to perform a practice block of five trials (which were identical to the experimental trials) to ensure that they fully understood the instructions. The trial sequence (see Fig. 3A) began with a central fixation point (+) 500 ms in duration. The fixation display was followed by a shape (a white square in version A, a lime rectangle in version B) that appeared in sequence at four locations for 2 sec (500 ms at each location). The shape marked one target location and three non-target locations. Once the last shape in the sequence was off, a black screen lasting 1, 5, 10 or 15 seconds, randomly selected, served as the delay interval. Then, the probe display was presented until the participant responded. The probe display contained two shapes (e.g., two white squares in version A or two lime rectangles in version B): one was the relevant shape, and the other was irrelevant. The relevant shape occupied the target location that was marked in the sequence, whereas the irrelevant shape occupied the non-target location that was not marked in the sequence.

Using a standard trial-and-error procedure, participants had to identify which of the 8 possible locations were the actual 4 target locations and then had to retain these identifications in memory. They selected the relevant shape by touching it on the screen with no time limit. The picture of a reinforcer (the outcome) followed the correct responses for 2.5 sec. Incorrect responses were followed by a blank screen that lasted the same time as the outcome presentation for the correct response. The next trial began after an additional interval of 500 ms.

The experiment consisted of 64 training trials grouped in two blocks of 32 trials each. The four target locations were presented 16 times as the locations occupied by the relevant shape in the probe display. The four non-target locations were also presented 16 times as the locations occupied by the irrelevant shape in the probe display.

Two versions of the spatial memory task were utilized. The low-attentional task was as previously described. The high-attentional task incorporated two primary changes to the low-attentional task. First, one shape in the sequence was printed in a red color, and children were told to tap their hand on the table as soon as the red shape came up (the secondary task). Second, in the probe display, apart from the relevant and irrelevant shapes such as in the low-attentional task, a second irrelevant shape was also presented in a target location that was not marked by the shape in the sequence of a particular trial (Fig. 3B). These changes made the task sufficiently difficult to avoid the task with the younger children. Thus, a group of twelve 5-year-old children along with another group of seventeen 7-year-old children performed the low-attentional task. A different group of fourteen 7-year-old children performed the high-attentional task only.

## Additional Information

**How to cite this article**: Esteban, L. *et al.* Spatial working memory is enhanced in children by differential outcomes. *Sci. Rep.* **5**, 17112; doi: 10.1038/srep17112 (2015).

## Figures and Tables

**Table 1:** Mean (and standard deviation) scores obtained on the Peabody Picture Vocabulary Test (PPVT-III) and the Automated Working Memory Assessment (AWMA).

	PPVT-III		AWMA		
	*Raw Score*	*Mental Age*	*STM_VS*	*WM_VS_R*	*WM_VS_P*
Low-attentional Task					
* 5-Year-Old-Group*	53.3 (13.4)	7.1 (1.5)	105.4 (17.3)	113.1 (16.2)	114 (17.1)
* 7-Year-Old-Group*	79.8 (9.8)	10.7 (1.6)	104.1 (13.2)	118.9 (9.7)	113.5 (10.9)
High-attentional Task					
* 7-Year-Old-Group*	85.2 (9.0)	11.9 (2.0)	108.6 (15.0)	108.9 (12.3)	105.0 (12.8)

Note. The results obtained in the verbal tests of the AWMA (listening and verbal recall) are not included. PPVT-III = Peabody Picture Vocabulary Test; AWMA = Automated Working Memory Assessment; STM_VS = Short Term Memory-Visuospatial (dot matrix test); WM_VS_R = Working Memory-Visuospatial Recall (spatial recall test); WM_VS_P = Working Memory-Visuospatial Processing (spatial recall test).
